# Clinical and Therapeutic Notes

**Published:** 1887-12-03

**Authors:** C. R. Illingworth


### CLINICAL AND THERAPEUTIC NOTES.

BY C. R. ILLINGWORTH, M.D., M.R.C.S.

DISEASES ASSOCIATED WITH DIMINUTION OF FIBRIN.

THE next group of diseases we have to consider embraces a very large number within its compass. It may be called the *Septicæmic* group, although many of the class are not septicæmic in origin. In these diseases the great feature is the diminution in the quantity of fibrin. The chief symptoms vary according to the origin of the diminution. In some a distinct dusky hue of the skin is observed; in others there are effusions of blood under the skin, the blood being so devoid of fibrin that it oozes through the capillaries; whilst in many, hæmorrhages from the nose and other parts lead to urgent complications in the course of the disease. Examples of the class are puerperal septicæmia and typhoid fever; but seeing that the diminution of fibrin is brought about in various ways, it may be well to submit the group to a process of division. Firstly, therefore, there are diseases due to a diminution of the fibrin-forming power of the blood from the absorption of decomposition products. Taking puerperal septicæmia (or pyæmia) as a type of this class, it is only necessary to refer to the morbid anatomy in a case of the kind to find it an established fact that "the blood is usually dark-coloured and imperfectly coagulated" (*Jones and Sieveking*). Other examples are found in pyæmia of surgical origin, such as were of frequent occurrence before the introduction of the antiseptic treatment of wounds.

Secondly, there are those due to the lodgement, growth, and development of specific germs in the system, which, by their depredations upon the oxygen in the corpuscles, diminish the fibrin-forming power of the blood very remarkably. Such are scarlet fever, diphtheria, measles, small-pox, hydrophobia, etc., etc. In some of these diseases this action upon the corpuscles is frequently intensified, and gives rise to a *malignant* form, characterised by a dusky or bluish hue of the skin, as in blue measles and malignant scarlet fever; and by hæmorrhage, as in petechial typhus fever and hæmorrhagic small-pox.

Thirdly and lastly, there are those diseased processes which very frequently supervene upon acute chest affections, such as bronchitis, pneumonia, and broncho-pneumonia, in which, from blocking of the respiratory tract due to abundant secretion or effusion of inflammatory products into the air-cells, there is the development of an asphyxiated condition. The symptoms presented are the following: The face and lips have a dusky hue, the respiration is rapid and laboured, and large moist sounds are audible to the bystander. With the diminished fibrin-forming power, a new and a dangerous process is initiated in the effusion of the watery elements of the blood into the respiratory tract. In such cases either the administration of full and frequently-repeated doses of iron, or the inhalation of oxygen, or both, are of immense service. I have repeatedly seen cases of the kind, with respirations amounting to ninety and one hundred in the minute, rapidly relieved and eventually cured with from one to five doses of the strong perchloride of iron every quarter of an hour. It will be remembered that quite recently an eminent Continental surgeon was saved, when similarly afflicted, by the daily administration of oxygen by inhalation. But oxygen is not a remedy which can be easily conveyed in general practice, nor is it one which the general practitioner would relish being called upon to manufacture upon short notice. Iron is enough, and iron is the remedy *par excellence* for all diseases which are associated with or characterised by a diminution in the fibrin of the blood. But the origin of the disease must also receive attention. In the first or purely septic class there are indications for the application of local cleansing agents, such as carbolic acid, bichloride of mercury, Condy's fluid, etc., to the infecting sources where such treatment is practicable Also for the administration of germicides, such as the biniodide of mercury, which may be prescribed in pills of the strength of one-sixteenth to one quarter of a grain three times a day. Alcoholic stimulants, such as brandy, wine, and wine whey, and concentrated fluid nutrients, are urgently needed in all these diseases. In cases of pneumonia occurring in persons who are the subjects of chronic alcoholism it is well known that the disease is apt to take a very rapidly fatal form. The reason for this is not far to seek. In persons addicted to the abuse of alcohol the blood is deficient in fibrin; hence the more ready supervention of the condition just described. In these cases stimulants are almost as essential as iron in treatment. When it is necessary, from the dry state of the tongue and the general febrile condition, to prescribe refrigerant salines along with the iron, an excellent combination will be found in a mixture of nitrate of potash and perchloride of iron well sweetened with chloric ether and glycerine.

The most marked cases of the production of an over-fluid condition of the blood—or, to speak more definitely, of the loss or great impairment of its fibrin-forming power—which I have ever seen, are those which occur in horses and other animals in some hot countries. In certain parts of South Africa a disease called "horse sickness" is very prevalent at certain seasons of the year, and is very fatal. It attacks horses, mules, goats, and oxen, but donkeys invariably escape. After suffering heavy losses in horse-flesh during the Zulu War, the Government secured a large number of asses for service in the northern parts of the Transvaal, and they enjoyed complete immunity from attacks of the disease. These hardy animals are also much used by gold diggers in their journeys to the Fields. During my term of office as a civilian surgeon attached to the Army Medical Department in South Africa in 1879, I had many opportunities of observing the course of the disease, and I dissected many horses and mules which had died of it. The blood becomes so fluid that it filters through the capillaries and lining membranes of the air-cells and bronchi, and the animal dies with the frothed-up fluid running out of its nostrils—literally drowned in its own blood. On dissection, the pleural cavities, pericardium, and bronchi are filled with serum, whilst the parenchyma of the lungs and the subcutaneous tissues are filled with the same fluid. In mules the subcutaneous tissue is more affected than in horses, giving rise to a thick-headed appearance, called *dikkop* by the Dutch—one of the names by which the disease is known. There are no signs of inflammatory action anywhere, although it is generally believed that the disease is of the nature of pleurisy. This deeply-rooted opinion, however, is founded in error. An examination of the mesenteric glands reveals the cause of the disease. They contain numbers of animalculæ which must have passed from the intestine in embryo form, and it is to their presence in the course of the circulation of the blood that we must ascribe the origin of the disease. The remedies which have been most successful are bleeding and purging, but in the vast majority they utterly fail even to relieve. I should think that iron and the biniodide of mercury would prove useful in conjunction with blood-letting to forty or fifty ounces. Animals which recover from the disease are known as "salted," and when duly certified as such, "salted" horses are much sought after, and highly valued. In that very readable book, "The Transvaal of To-day," by Mr. Alfred Aylward, a graphic account is given of the writer's experience with "salted" horses. He says: "My first 'salted' animal, Peter, is still alive and well, and valued at 100 guineas, after having travelled, during my ownership of him, in peace and war, nearly three thousand miles, sometimes being as long as nineteen hours without having the saddle for one moment off his back." And "good animals, guaranteed but slow, used to being shot over, can always be obtained at from £35 to £50; but fast, lively, and spirited trained 'salted shooting horses' are worth from £70 to £150; and no price can be too high for some of the good, clever, and faithful creatures I have known."

